# First Report of *Trichinella britovi* in Serbian Domestic Pigs Linked to Two Human Outbreaks

**DOI:** 10.3390/vetsci13070717

**Published:** 2026-07-21

**Authors:** Ivana Mitic, Milos Korac, Ewa Bilska-Zając, Jasna Kureljusic, Ana Vasic, Dragan Vasilev, Sasa Vasilev

**Affiliations:** 1 Institute for the Application of Nuclear Energy, University of Belgrade, 11000 Belgrade, Serbia; 2 Institute of Public Health of Serbia “Dr. Milan Jovanovic Batut”, 11000 Belgrade, Serbia; 3 Faculty of Medicine, University of Belgrade, 11000 Belgrade, Serbia; 4 Hospital for Infectious and Tropical Diseases, Clinical Center of Serbia, 11000 Belgrade, Serbia; 5 Department of Parasitology and Invasive Diseases, National Veterinary Research Institute in Poland, 24-100 Pulawy, Poland; 6 Scientific Institute of Veterinary Medicine of Serbia, 11000 Belgrade, Serbia; 7 Faculty of Veterinary Medicine, University of Belgrade, 11000 Belgrade, Serbia

**Keywords:** *Trichinella britovi*, outbreak, One Health, Serbia

## Abstract

Sporadic cases of trichinellosis have occurred almost every year in Serbia. The success of trichinellosis control in the country depends on effective communication among medical professionals who discover a case of trichinellosis, the Public Health Service, and the Veterinary Service, in line with the One Health concept. The collaborative approach, supported by a robust reporting and monitoring system, has been essential for identifying infection sources and preventing further spread. However, in March 2023, the National Reference Laboratory for Trichinellosis (NRLT) received reports of hospitalized patients with suspected trichinellosis, signaling the possibility of two outbreaks based on their socio-epidemiological history. The investigation revealed separate trichinellosis outbreaks occurred in Grocka, Belgrade municipality, and Dolovo, Pancevo municipality, with pork identified as the source of infection. Serological tests confirmed the presence of *Trichinella*-specific antibodies in the patients, including their family members and friends. A total of four patients required hospitalization. In accordance with regulations, veterinary inspectors took dried meat and meat products samples and *Trichinella* larvae were detected using the digestion method. The entire amount of dried meat and meat products was subsequently destroyed. Multiplex PCR confirmed the identification of *Trichinella britovi* at the species level in both outbreaks. These findings represent the first documented cases of *T. britovi* infection in domestic pigs in Serbia. The outbreak data once again highlight that inadequate animal husbandry practices, certain human behaviors, and a lack of awareness about the risks continue to be major contributors to the persistence of trichinellosis in Serbia.

## Simple Summary

Trichinellosis is a disease that people can get by eating raw or undercooked meat containing infective *Trichinella* larvae. Although the disease is now less common in Serbia, isolated cases continue to occur every year. In 2023, two separate outbreaks were identified in the areas of Grocka and Dolovo after several people became ill and some required hospital treatment. Investigations by medical and veterinary authorities showed that the infections were linked to pork and pork products from domestic pigs. Laboratory testing confirmed the infection in affected people and detected the parasite in the meat products. The contaminated meat was removed and destroyed to prevent further infections. The study also identified, for the first time in Serbia, a parasite species (*Trichinella britovi*) that had not previously been documented in domestic pigs in the country. These outbreaks demonstrate the importance of close cooperation between human and animal health services for rapid detection and control of disease. The findings also highlight that poor animal management practices, unsafe food preparation, and limited awareness of infection risks continue to contribute to the persistence of trichinellosis in Serbia.

## 1. Introduction

Parasitic nematodes classified into the genus *Trichinella* are distributed worldwide, can infect many different animal species and represent causative agents for serious and important zoonotic disease trichinellosis [1,2]. *Trichinella* spp. infections in animals constitute an important foodborne zoonotic risk, as human infection occurs following the consumption of raw or insufficiently cooked meat from infected animals containing viable larvae. Although trichinellosis is well controlled in Serbia, *Trichinella* infection continues to be an animal husbandry problem and human health threat. Trichinellosis is an endemic and mandatory reportable disease in Serbia. In Serbia, the implementation of the One Health concept relies on cooperation and the timely exchange of comprehensive information among all relevant parties. When physicians at the CITD diagnose a case of trichinellosis, they report it to the appropriate City Public Health Institute, which then alerts the Veterinary Inspection. The Veterinary Inspection notifies relevant veterinary specialists or scientific institutes. Simultaneously, the NRLT and the NRL for the detection of *Trichinella* in domestic and wild animal meat are also notified. It is noteworthy that both NRLTs are situated within the INEP, and the experts from INEP collaborate within both NRLTs [3,4].

Training programs for veterinarians have been conducted at the Faculty of Veterinary Medicine and the Institute of Meat Hygiene and Technology. The NRLT actively contributes by organizing courses for doctors and veterinarians, publishing articles in professional and scientific journals, and regularly conducting proficiency testing (PT). These initiatives aim to enhance the expertise of laboratories performing *Trichinella* larvae examinations, and to improve the competence of their personnel. According to the 2022 Serbian regulation, participation in *Trichinella* PT organized by the NRLT is mandatory [4]. In alignment with the One Health approach, the Veterinary Directorate of the Ministry of Agriculture, Forestry, and Water Management, as the legislative body, has collaborated with the NRLT to develop educational brochures for hunters and pig breeders. This comprehensive approach represents the collective efforts of all stakeholders involved in the One Health concept, fostering the connection and collaboration among them. Sporadic family outbreaks predominantly occur during the seasons of domestic pig slaughtering and wild boar hunting, often linked to the consumption of meat products that lack veterinary inspection [3,4]. Similar seasonal patterns are observed in neighboring countries such as Bulgaria and Romania [5,6]. The epidemiology of trichinellosis in Serbia involves detected species *Trichinella spiralis* (*T. spiralis*) and *Trichinella britovi* (*T. britovi*) wherein as the hosts of *T. britovi* only wild boars have been reported. Trichinellosis cases occur sporadically, resulting from outbreaks where individuals fail to implement preventive measures, such as bringing to inspection by authorized veterinarians home-produced meat and/or acquiring/receiving meat products (e.g., fermented and dry sausages, ham, and raw sausages for roasting) from sources they believe to be safe. The primary method of preventing trichinellosis is the examination of slaughtered pigs and susceptible animals intended for human consumption for the presence of Trichinella larvae combined with information campaigns. Despite notable outbreaks in Serbia over the past decade, the incidence of trichinellosis among humans has displayed a declining trend. This decline underscores the importance of food safety programs, as most positive cases involve individuals who consumed domestic swine and wild boar meat without proper veterinary control [4]. This study aimed to present data from two notable trichinellosis outbreaks of 2023, one in the Belgrade district and the other in a village in the South Banat district, and to discuss and identify the key challenges that affect the successful and effective control of *Trichinella* infections.

## 2. Materials and Methods

Epidemiological, serological and parasitological data were collected from two trichinellosis outbreaks in 2023 and analyzed.

**Figure 1:**
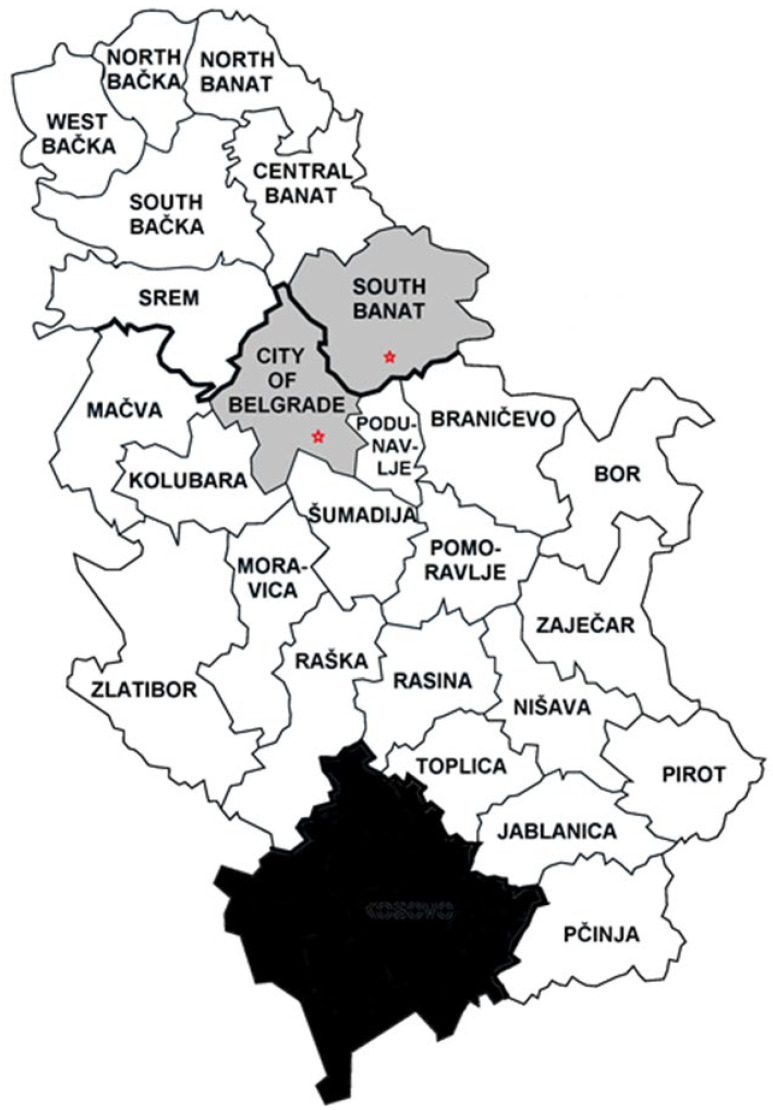
Districts in Serbia affected by outbreaks in 2023: https://en.wikipedia.org/wiki/Districts_of_Serbia#/media/File:Districts_of_Serbia.png accessed on 20 May 2026. The map highlights the Belgrade and South Banat Districts in gray. Red stars indicate the locations of outbreaks caused by the consumption of untested pork infected with *Trichinella britovi*.

Epidemiological data and timeline of the outbreak: Trace-back studies and outbreaks investigation: On 10 March 2023, the Veterinary Inspection in Belgrade contacted the Scientific Institute of Veterinary Medicine of Serbia (IVMS). The reason for this communication was the notification from the City Institute for Public Health Belgrade, informing them about nine suspected (one of them was in the hospital) trichinellosis cases in Grocka (44°40′12″ N; 20°43′02″ E) (Figure 1). The inspection urgently requested analysis of the dry meat and meat products that were discovered. The IVMS informed the NRLT and collaborated on the subsequent steps. Following the advice of the NRLT, the IVMS performed artifical digestion using gold standard magnetic stirrer method (MSM) [7,8,9] and multiplex PCR to detect Trichinella species, as described in work of Cvetkovic et al. [10], and submitted the Trichinella larvae preserved in alcohol to the NRLT for confirmation of species identification, as required by regulations.

At the same time, the Clinic for Infectious and Tropical Diseases, University Clinical Center of Serbia (CITD) in Belgrade, alerted NRLT INEP about two patients who were hospitalized with symptoms suggestive of trichinellosis. Based on the patients’ socio-epidemiological history, it was determined that there were two outbreaks. The second outbreak site (3 individuals involved) occurred in the village of Dolovo (44°54′02″ N; 20°52′37″ E) (Figure 1), within the municipality of Pancevo, South Banat district. The veterinary inspection of the Municipality of Pancevo requested a meat analysis for the presence of *Trichinella* larvae. This analysis was performed at the Veterinary Specialized Institute Pancevo using MSM, which subsequently informed the NRLT and provided samples of sausage and kulen—traditional fermented meat products for larvae isolation and species determination.

Serological testing of patients: On the first day of hospitalization, blood samples were collected from two patients (one from Dolovo outbreak and one from Grocka) suspected of trichinelloisis, which corresponded to approximately four weeks after the consumption of infected meat products. Serodiagnosis was conducted at NRLT INEP using two methods: IFA using “FITC *Trichinella spiralis* Antibody Detection Kit” from INEP, Serbia, and an in-house ELISA test “INEP-*Trichinella* ELISA test ”. The testing procedures followed previously described protocols [11,12]. In the IFA test, serum samples showing anti-*Trichinella* antibody titers of ≥1:40 and displaying a bright, apple-green signal on the cuticle and inside the stichocytes of the parasite muscle larvae were considered seropositive. For the ELISA test, the index value was calculated using the formula: index = [(OD mean duplicate sample—OD mean duplicate blank)/(OD mean duplicate negative control—OD mean duplicate blanks)]. Serum samples with an index value of ≥3.7 were classified as seropositive. The optimal cut-off value for the index was determined to be 3 ± 2 standard deviations (SDs), and index values falling within the range of the cut-off (2.3–3.7) were considered in the gray zone.

Parasitological examination of the swine meat products: The veterinary inspectors provided samples of dry fermented sausages, which were analyzed to determine the presence of *Trichinella* spp. muscle larvae using the recommended MSM, in accordance with ISO standard and EU and Serbian regulations [7,8,9]. After digestion, larvae were collected, and the worm burden was quantified as larvae per gram (LPG) of tissue.

Molecular identification and confirmation of *Trichinella* species: Species identification of *Trichinella* larvae was conducted using multiplex PCR analysis with a test recommended by the European Union Reference Laboratory (EURLP, Istituto Superiore di Sanitá in Rome, Italy) [13]. The isolated larvae were reanalyzed by multiplex PCR in Poland (by the same EURLP method), and sequencing was performed as previously described [14].

## 3. Results

Epidemiological investigation: An investigation confirmed a trichinellosis outbreak in the Grocka municipality of Belgrade, traced to the consumption of fermented pork sausages. The owner of the meat products stated he had purchased a pig for slaughter; however, veterinary authorities were unable locate the alleged seller. There was no documentation or evidence that the meat has been tested for the presence of *Trichinella* larvae. Meat products were consumed by the owner, his family, and friends resulting in nine cases of trichinellosis, four of whom required hospitalization. Based on the available information, illegal pig breeding and unregulated pork trade are suspected. Around the same time, another outbreak occurred in the village of Dolovo, within the Pancevo municipality, South Banat district. The source of infection was a traditional meat product prepared from uncontrolled backyard pigs raised for personal consumption. Only the couple who raised the pigs was infected. The woman was hospitalized, and only then did they recognize that the husband had also been experiencing symptoms, which had previously gone unnoticed. The couple confirmed that the products were made from their own pigs, and that one of the slaughtered animals, specifically the one used for these products, had not undergone any veterinary inspection.

Clinical investigation: In the first patient from the Grocka outbreak, the symptoms started around March 1st with diarrhea and vomiting which lasted for 2 days, followed by swelling of the face and redness and swelling of the eyes, and pain in the muscles of the lower legs. The patient reported to the CITD in Belgrade, where hematological and biochemical findings were indicative of trichinellosis. Analyses revealed a peripheral blood leucocyte count of 10.8 × 10^9^/L with an elevated eosinophil percentage of 22.9%, a C-reactive protein level of 23.5, and an increased creatine kinase value of 670.

Around March 1st, a patient from the Dolovo outbreak developed diarrhea that lasted for 3 days followed by a fever of up to 38 °C. On the seventh day from the onset of symptoms, large swelling of the face and eyelids developed, with difficulty in breathing when lying down and rashes on the skin, as well as swelling of the extremities and severe pain in the muscles. The patient was examined at the CITD in Belgrade, where hospitalization was suggested.

Immunoserological analysis: Serological testing performed at the NRLT INEP using both IFA and ELISA methods confirmed the presence of anti-*Trichinella* antibodies in two patients hospitalized at the CITD in Belgrade. In the case linked to the Grocka outbreak, the patient had an anti-*T. spiralis* antibody titer of 1:640 in the IFA test and a positive ELISA result with an index value of 3.9. The patient associated with the Pancevo outbreak also had an antibody titer of 1:640 and a positive ELISA result with an index of 5.8.

Parasitological analysis: Analysis done in IVMS showed the presence of *Trichinella* larvae and the larval burden in sausage and dried meat products confiscated in the Grocka outbreak was 0.4 LPG and 2 LPG respectively. After performing the MSM at the NRLT, the presence of *Trichinella* larvae was verified. In the Dolovo outbreak, MSM at the NRLT verified the presence of *Trichinella* larvae, with larval burden of 100 LPG in pork sausages and 23 LPG in the fermented meat product, kulen. All seized meat from both outbreaks was destroyed by the inspection in accordance with the current regulations and legal requirements.

Molecular identification and confirmation of *Trichinella* species: Multiplex PCR analysis of larvae from the Grocka outbreak (conducted by the IVMS and NRLT) and the Dolovo outbreak (performed in the NRLT) identified the species involved in both cases as *T. britovi.* The PCR results showed a characteristic two-band pattern at 127 bp and 253 bp, which is indicative of *T. britovi* in multiplex PCR. This marks the first recorded detection of *T. britovi* in domestic pig meat in Serbia. As part of the bilateral collaboration, the isolated larvae were reanalyzed by multiplex PCR in Poland, and sequencing was initiated to investigate the potential epidemiological link between the two outbreaks. Independent multiplex PCR analysis again confirmed *T. britovi* as the etiological agent in both outbreaks. However, sequencing analysis could not be completed because the limited number of available larvae and the insufficient quality and quantity of recoverable DNA precluded the generation of reliable sequence data. Consequently, sequence-based confirmation and assessment of a possible epidemiological relationship between the outbreaks could not be achieved.

Ethical statement: The study was planned, conducted, and reported in accordance with the principles outlined in the Declaration of Helsinki (2013 revision). Ethical approval was not required, as the data presented were obtained as part of routine diagnostic procedures and outbreak surveillance carried out by NRLT INEP.

## 4. Discussion

The occurrence of two trichinellosis outbreaks in 2023 highlights the persistent challenge of controlling *Trichinella* infections, placing Serbia among the Balkan countries that continue to be regarded as endemic for trichinellosis, despite extensive prevention efforts.

Trichinellosis is still prevalent in Serbia due to various factors: negligent meat owners who fail to give the sample for meat inspections, occasional professional errors that involve the continued use of the compression method, and inspection by technicians instead of veterinarians in certain locations contrary to regulations [15].

However, errors during meat inspection, including the involvement of unqualified personnel following outdated regulations, the use of inadequate testing methods, inappropriate sample selection, or insufficient sample quantity for inspection, can lead to trichinellosis outbreaks [16]. Non-compliance with the new regulations [9] that for the compression method requires a minimum of 1 g, including 56 sample cuts to be inspected, while the previous rulebook requested only 28 cuts, could be one of the most serious reasons for false results during meat inspection.

In 2011, *Trichinella* spp. muscle larvae were detected in 523 inspected swine carcasses (prevalence 0.026%), with the numbers steadily declining over following years to 93 infected animals in 2020. That year, the infection rate in domestic swine was 0.0037%, reflecting the successful maintenance of low prevalence during the observed period [4]. In Serbia, detection of *Trichinella* spp. larvae is mandatory at slaughterhouses, with the digestion method required for meat inspection. For domestic swine meat intended for market, both the digestion method and trichinelloscopy may be used, whereas wild boar meat must be tested exclusively by digestion [4,15]. Improvements in pig rearing systems and the implementation of control measures have eliminated *Trichinella* infection in pigs from controlled housing systems, with no positive animals reported from such farms [4,17,18,19]. Nowadays, all *Trichinella*-positive pigs originate from backyard holdings with uncontrolled housing conditions [4].

Until now, molecular analyses performed on *Trichinella* isolated from infected pig meat (at INEP, and EURLP ISS, Rome, Italy) revealed the presence of only *T. spiralis* in domestic pigs in Serbia [4], out of four *Trichinella* species (*T. spiralis*, *T. britovi*, *T. pseudospiralis* and *T. nativa*) recognized in Europe [2]. *T. britovi* was confirmed in samples of wild boar meat, and this *Trichinella* species caused a huge outbreak in Serbia in 2016 after consumption of wild boar meat [20,21].

This study reports the first detection of *T. britovi* infection in domestic pigs in Serbia. Species identification was independently confirmed by multiplex PCR performed in two laboratories, based on the characteristic two-band pattern (127 bp and 253 bp). As part of a bilateral collaboration, sequencing analysis was undertaken to further investigate a possible epidemiological link between the two outbreaks. However, due to the limited number of available larvae and the insufficient quality and quantity of recoverable DNA, reliable sequence data could not be obtained. Therefore, although concordant multiplex PCR results obtained independently in two laboratories strongly support the identification of *T. britovi*, the lack of sequence-based confirmation and phylogenetic analysis represents a limitation of the present study and precluded further investigation of a possible epidemiological relationship between the outbreaks.

This study reports the first detection of *T. britovi* infection in domestic pigs in Serbia. The presence of *T. britovi* in domestic pigs could indicate a potential spillover from the sylvatic cycle, as this *Trichinella* species had previously been reported only in wild carnivores in Serbia [10,22,23]. In the sylvatic cycle, the golden jackal (*Canis aureus*), a rapidly expanding and synanthropic species, has been identified as a key host, carrying *T. britovi*, *T. spiralis*, or mixed infections of both [23]. *T. britovi* was also found in red foxes (*Vulpes vulpes*) and wolves (*Canis lupus*) in 2009 [10], alongside the already confirmed presence of *T. spiralis* in the wildlife of Serbia [24].

In our study, the infected pigs originated from two unrelated farms, not connected in any way, and in both cases the backyard pigs were kept without all the necessary hygiene measures, adequate biosecurity and proper management. The meat was not submitted for parasitological examination, and in both households human infections occurred.

Consumption of uninspected and undercooked meat from backyards and locally slaughtered domestic pigs on private properties is recognized as a major risk factor and a main source of human *Trichinella* infections [4]. In these 2023 outbreaks, infections affected both the individuals involved in meat processing and their families and friends. On the contrary, pork from farms with controlled housing conditions, where proper biosecurity, rearing and control measures are implemented, poses no risk for human infection [4].

During 2011–2020 period, a total number of 699 cases of trichinellosis, on average 69.9 per year, without deaths, and the annual incidence of trichinellosis varied from 2.68 to 0.16 per 100.000 inhabitants, has been reported in Serbia, mostly due to inadequate awareness of the risk from consumption of untested wild boar and backyard and free ranging pigs’ meat [4]. Epidemiological investigations of trichinellosis outbreaks in Serbia revealed that in 88.09% of the outbreaks the source of infection was raw or undercooked pork meat or meat products, in 2.38% it was horse meat and in 9.52% it was wild boar meat [4]. In recent years, trichinellosis outbreaks have become less frequent in Serbia [3]. According to data from the past five years (2020–2024), only seven small family-related outbreaks were reported, with no sporadic cases or outbreaks registered in 2021 and 2024.

The clinical course of trichinellosis may vary by *Trichinella* species. Infections with *T. spiralis* are generally more severe than those with *T. britovi*, showing longer-lasting IgG responses, higher CK levels, and more pronounced intestinal symptoms [25,26]. In contrast, *T. britovi* infections tend to be milder with a longer incubation period [27]. Accordingly, during a *T. britovi* outbreak in Italy, patients experienced a mild form of the disease with short-term symptoms and did not require hospitalization [28]. However, the severity of trichinellosis is influenced not only by the *Trichinella* species involved, but also by the number of ingested larvae, as well as the host’s gender, age, and immune status [29]. During the 2016 outbreak in Serbia, caused by *T. britovi*, nine out of 111 patients developed cardiac complications. Parasitological analysis showed a larval burden of 0.18 LPG in dry meat and 0.87 LPG in sausages [21]. In two of the outbreaks described here, patients infected with *T. britovi* exhibited allergic symptoms such as eyelid and facial swelling, along with difficulty breathing, with one case from the Pancevo outbreak initially resembling an anaphylactic reaction. In the Pancevo outbreak, the high larval burden of approximately 100 LPG in pork sausages and 23 LPG in kulen could account for the anaphylactic-like reaction observed in the patient. It is likely that the intensity of infection, reflected by the number of ingested larvae, played a key role in the severity of the clinical presentation.

## 5. Conclusions

This study reported for the first time in Serbia two trichinellosis outbreaks caused by consumption of meat from domestic pigs infected with *T. britovi*. The study also highlights that risk factors are still present in Serbia and that they influence the existence of human cases. In Serbia the success in the field of *Trichinella* infection control is evident, but when backyard pigs are raised in uncontrolled conditions and the animals are not subjected to veterinary inspection meat and meat products can be a source of infection.

## Data Availability

The original contributions presented in this study are included in the article. Further inquiries can be directed to the corresponding author.
